# Efficacy and safety of neoadjuvant chemoradiotherapy versus neoadjuvant chemotherapy in locally advanced esophageal cancer: An updated meta-analysis

**DOI:** 10.1097/MD.0000000000036785

**Published:** 2024-01-19

**Authors:** Zhanpeng Liang, Ting Chen, Wenxia Li, Huiqin Lai, Luzhen Li, Jiaming Wu, Huatang Zhang, Cantu Fang

**Affiliations:** a Department of Oncology, Zhongshan Hospital of Traditional Chinese Medicine Affiliated to Guangzhou University of Traditional Chinese Medicine, Zhongshan, China.

**Keywords:** locally advanced esophageal cancer, meta-analysis, neoadjuvant chemoradiotherapy, neoadjuvant chemotherapy

## Abstract

**Background:**

Currently, the optimal treatment for neoadjuvant therapy for locally advanced esophageal cancer is not clear, and there is no evidence that neoadjuvant chemoradiotherapy (nCRT) is superior to neoadjuvant chemotherapy (nCT). Due to the publication of new clinical trials and defects in previous meta-analyses, we conducted an updated meta-analysis to evaluate the efficacy and safety of nCRT and nCT.

**Methods:**

The following databases were searched for studies: PubMed, EMBASE, and Cochrane library (updated to April 22, 2023). All randomized trials comparing nCRT with nCT in locally advanced esophageal cancer met the inclusion criteria. Data were analyzed using Review Manager 5.4.1 (Cochrane collaboration software). Primary outcomes assessed from the trials included overall survival (OS), progression-free survival (PFS), pathological complete response (pCR), R0 resection rate, postoperative complications, postoperative mortality, and grade 3 or higher adverse events (3 + AEs).

**Results:**

This systematic review and meta-analysis included 7 randomized controlled studies involving 1372 patients (686 receiving nCRT and 686 receiving nCT). Compared with nCT, nCRT significantly improved OS (HR = 0.80; 95% CI: 0.68–0.94), PFS (HR = 0.78; 95% CI: 0.66–0.93), pCR (OR = 13.00; 95% CI: 7.82–21.61) and R0 resection (OR = 1.84; 95% CI: 1.32–2.57), but was associated with higher postoperative mortality (OR = 2.31; 95% CI: 1.26–4.25) and grade 3 + AEs (OR = 2.21; 95% CI: 1.36–3.58). There was no significant difference in postoperative complications between nCRT and nCT (OR = 1.15; 95% CI: 0.82–1.61). Subgroup analysis showed significant survival benefit in squamous cell carcinoma (HR = 0.80; 95% CI: 0.68–0.98), but not in adenocarcinoma (HR = 0.80; 95% CI: 0.63–1.08).

**Conclusions:**

Our meta-analysis found superior efficacy associated with nCRT compared with nCT in both tumor regression and prolonged survival, but increased the risk of postoperative mortality and grade 3 + AEs. Esophageal squamous cell carcinoma was more likely to benefit from nCRT than esophageal adenocarcinoma in the term of OS.

## 1. Introduction

Esophageal cancer, the sixth leading cause of cancer death in the world, is a disease with a poor prognosis^[1]^ Surgery is a common treatment for locally advanced resectable (or potentially resectable) esophageal cancer, but the results are not satisfactory. The 5-year survival rate after surgery alone is very low, and most patients have local recurrence or distant metastasis after surgery.^[2,3]^ In order to further improve the survival of patients with locally advanced esophageal cancer, a variety of new treatment programs continue to emerge. Based on OEO2^[4]^ and CROSS trials,^[5,6]^ neoadjuvant chemotherapy (nCT) and neoadjuvant chemoradiotherapy (nCRT) have replaced surgical treatment alone as the standard treatment for locally advanced esophageal cancer. Whether nCRT is better than nCT is always a concern. Previous randomized clinical trials comparing the effects of nCRT and nCT in esophageal cancer have found that chemoradiotherapy could improve local control but did not reduce the risk of distant metastasis.^[7,8]^ Meanwhile, nCRT may increase the risk of postoperative complications and death, offsetting some of the benefit trend.^[9,10]^ Thus, chemoradiotherapy provided a higher rate of complete histopathological response, but no statistically significant improvement in survival. Due to insufficient data provided by previous randomized trials, some previous meta-analyses included cohort studies, leading to low levels of evidence, unreliable conclusions and some major defects.^[11]^ Moreover, meta-analyses of randomized trials do not support survival benefits of nCRT^[12–14]^]. So far, the jury is still out on whether to add additional radiation therapy. However, there is no evidence that nCRT is superior to nCT. In recent years, several important trials have been published that promise to change the current treatment landscape.^[15,16]^ Therefore, a rigorous and meta-analysis is necessary. We conducted a meta-analysis focusing only on randomized controlled trials to compare the efficacy and safety of nCRT versus nCT in locally advanced esophageal cancer.

## 2. Methods

This study was registered in the PROSPERO database (CRD42023423351) and was conducted according to the preferred reporting project for systematic review and meta-analysis (PRISMA) statement.^[17]^ The purpose of this study was to compare the efficacy and safety of nCRT and nCT in locally advanced esophageal cancer.

### 2.1. Eligibility criteria

The study was independently screened by 2 investigators. Inclusion criteria used to select studies in this meta-analysis were: patients with a cytological or pathological diagnosis of esophageal cancer, patients with a mean age >18 years, and prospective Phase II or III randomized trials comparing nCRT with nCT. Resectable (or potentially resectable) locally advanced esophageal cancer. Studies reporting at least one of the following outcomes: overall survival (OS), progression-free survival (PFS), pathological complete response (pCR), R0 resection rate, postoperative complications, postoperative mortality, and grade 3 or higher adverse events (3 + AEs).

Exclusion criteria: inoperable advanced esophageal cancer. Non-randomized controlled studies, basic studies, retrospective studies, case reports, duplicate publications, and studies for which relevant data cannot be extracted.

### 2.2. Literature search

The following databases were searched for studies: PubMed, EMBASE, Wei of science, and Cochrane library (the last search was dated April 24, 2023). The keywords or corresponding grid terms applied to Literature retrieval are: esophageal cancer, neoadjuvant, chemoradiotherapy, chemotherapy, surgery. The detailed search strategy is as described in the eMethods in the Supplement, http://links.lww.com/MD/L237. No language or date restrictions were applied to the searches. If overlapping data exists, the most complete and updated report is selected for inclusion in this meta-analysis. Manually review references from all eligible studies to find other relevant studies.

### 2.3. Study selection and data extraction

Two experienced investigators independently screened records for eligibility. Differences were resolved by consulting a third investigator. Browse titles and abstracts to complete an initial selection, followed by a full review of potentially eligible articles and a selection of eligible articles based on pre-established criteria. Extract data using a pre-specified data collection form. Extracted data included baseline characteristics, sample size, interventions, number of assessable patients, OS, PFS, pCR, R0 resection rate, postoperative complications, postoperative mortality and grade 3 + AEs, etc. Two investigators independently extract relevant data and resolve differences by consulting a third investigator. When multiple articles contained overlapping patient series, we prioritized extraction of outcome data from the primary articles with the largest sample size for early outcomes and the articles with the longest follow-up duration for late outcomes.

### 2.4. Risk of bias

Two investigators independently used the Cochrane Collaboration tool to assess the quality of included trials in the following areas: random sequence generation, assignment hiding, blinding, incomplete results data, and selective results reporting.^[18]^ Any differences in quality assessment were resolved by consulting a third investigator.

### 2.5. Statistical analysis

Data were analyzed using Review Manager 5.4.1 (Cochrane Collaboration Software). Outcomes of interest included OS, PFS, pCR, R0 resection rate, postoperative complications, postoperative mortality and 3 + AEs, which were either extracted directly from the article or calculated.^[19]^ pCR, R0 resection rate, postoperative complications, postoperative mortality and grade 3 + AEs were reported as odds ratio (OR), and PFS and OS were reported as hazard ratio (HR) with corresponding 95% confidence interval (95%CI). *P* < .05 was considered statistically significant. The I² statistic was applied to evaluate heterogeneity of the outcome. When the heterogeneity is low (I^2^ < 50%), the fixed effects model was used. Otherwise, the random effects model was used.^[20,21]^ The potential of publication bias was investigated by visually inspecting funnel plots for asymmetry and with the Egger regression test.^[22]^ Sensitivity analysis was performed by removing one study at a time to determine the association of individual studies with meta-analysis results. Subgroup analyses of histological type and baseline characteristics were planned to perform.

Statement: Our meta-analysis does not address the subject life, health, dignity, privacy, and other related issues. All analyses were based on previous published studies, thus no ethical approval or patient consent was required.

## 3. Result

### 3.1. Study identification and characteristics

A total of 2313 articles were retrieved from PubMed, EMBASE and Cochrane library. After removing 354 duplicates, 1919 were eliminated by reading the title and abstract. 40 full-text articles were reviewed. Finally, this meta-analysis included 7 randomized controlled trials^[7,8,15,16,23–25]^ involving 1372 patients. A PRISMA flow chart describing study identification and selection is shown in eFig. 1 in the Supplementary, http://links.lww.com/MD/L226. 6 trials were fully published reports,^[7,8,15,23–25]^ and 1 trial was only published in abstract form.^[16]^ 2 studies included only esophageal adenocarcinoma.^[7,23]^ 4 studies included only esophageal squamous cell carcinoma.^[15,16,24,25]^ 1 study included adenocarcinoma and squamous cell carcinoma^[8]^ (Table 1).

**Table 1:** Characteristics of included studies.

Author year	Accrual period	Country	Study design	Patient characteristics	Sample size	Tumor stage	Intervention	Control
Nygaard, 1992	1983.01–1988.01	Norway	RCT	ESCC	97	T3-4NXM0	DDP + bleomycin + radiotherapy(35Gy)	DDP + bleomycin
Cao, 2009	1991.02–2000.12	China	RCT	ESCC	237	II-IV	DDP + 5-FU + mitomycin+radiotherapy(40Gy)	DDP + 5-FU + mitomycin
Burmeister, 2011	2000.11–2006.12	Australia	RCT	EAC	75	T3-4NXM0	DDP + 5-FU + radiotherapy(35Gy)	DDP + 5-FU
Stahl, 2017	2000.11–2005.12	Germany	RCT	EAC	119	T3-4NXM0	DDP + 5-FU + folinic acid + radiotherapy(30y)	DDP + 5-FU + folinic acid
von Döbeln, 2019	2006-2013	Sweden	RCT	EAC + ESCC	181	T1N1 or T2–3N0-1M0-1a	DDP + 5-FU + radiotherapy(40y)	DDP + 5-FU
Kato, 2022	2012.12–2018.07	Japan	RCT	ESCC	399	Ib-III (Except T4)	DDP + 5-FU + radiotherapy(41.4y)	DDP + 5-FU
Tang, 2023	2017.01–2018.12	China	RCT	ESCC	264	cT3-4aN0-1M0	DDP + paclitaxel + radiotherapy(40y)	DDP + paclitaxel

DDP = cisplatin, EAC = esophageal adenocarcinoma, ESCC = esophageal squamous cell carcinoma, pCR = pathologic complete response, RCT = randomized controlled trial, 5-FU = 5-fluorouracil.

### 3.2. Risk of bias assessment

Since all studies included in the meta-analysis were randomized, selection and attrition bias were minimized. As expected, no mention of blindness was made in the included trials. However, there may be potential for some selection bias, owing to allocation concealment was not explicitly mentioned in some of the studies (eFig. 2–3 in the Supplementary, http://links.lww.com/MD/L227, http://links.lww.com/MD/L228).

### 3.3. Meta-analysis

#### 3.3.1. Overall survival.

**Figure 1.**
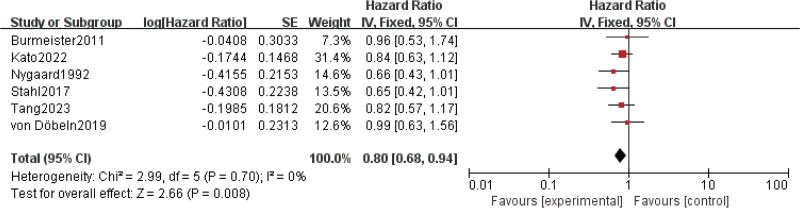
Assessment of Overall survival. Abbreviations: The diamond indicates best estimate of the true (pooled) outcome (with width indicating 95% CI); experimental stands for neoadjuvant chemoradiotherapy; control stands for neoadjuvant chemotherapy. Since there is no heterogeneity, a fixed-effects model is used.

Results for OS came from 6 studies^[7,8,15,16,23,24]^ involving a total of 1135 patients. The date of OS for 1 trial was difficult to extract from the Kaplan–Meier curve, so the OS of this study was excluded [24]. The results showed that compared with nCT, nCRT was associated with further improving OS and reducing the risk of death by 20% (HR = 0.80; 95% CI: 0.68–0.94; *P* = .008) (Fig. 1). No significant heterogeneity was found among the trials (Chi^[2]^ = 2.99; df = 5 [*P* = .70]; I^2^ = 0%).

#### 3.3.2. Progression-free survival.

**Figure 2.**
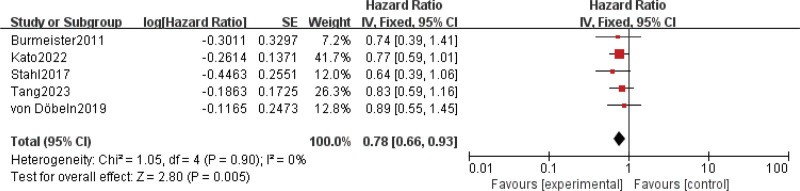
Assessment of Progression-free survival. Abbreviations: The diamond indicates best estimate of the true (pooled) outcome (with width indicating 95% CI); experimental stands for neoadjuvant chemoradiotherapy; control stands for neoadjuvant chemothrapy. Since there is no heterogeneity, a fixed-effects model is used.

Results for PFS were extracted from 5 studies^[7,8,15,16,23]^ involving a total of 1038 patients. The result showed that nCRT was associated with superior PFS compared with nCT, and reduced the risk of disease progression by 22% (HR = 0.78; 95% CI: 0.66–0.93; *P* = .005) (Fig. 2). No significant heterogeneity was found among the trials (Chi^[2]^ = 1.05; df = 4 [*P* = .90]; I^2^ = 0%).

#### 3.3.3. Pathological complete response.

**Figure 3.**
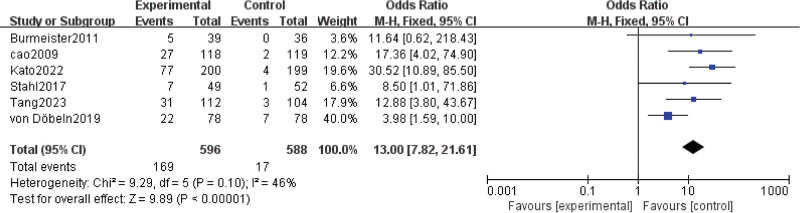
Assessment of Pathological complete response. Abbreviations: The diamond indicates best estimate of the true (pooled) outcome (with width indicating 95% CI); experimental stands for neoadjuvant chemoradiotherapy; control stands for neoadjuvant chemotherapy. Since there is moderate heterogeneity, a fixed-effects model is used.

Results for pCR were extracted from 6 studies^[7,8,15,16,23,25]^ involving a total of 1184 patients. The result showed that nCRT significantly improved pCR (OR = 13.00; 95% CI: 7.82–21.61; *P* < .00001) (Fig. 3). Moderate heterogeneity was found among the trials (Chi^[2]^ = 9.29; df = 5 [*P* = .10]; I^2^ = 46%).

#### 3.3.4. R0 resection rate.

**Figure 4.**
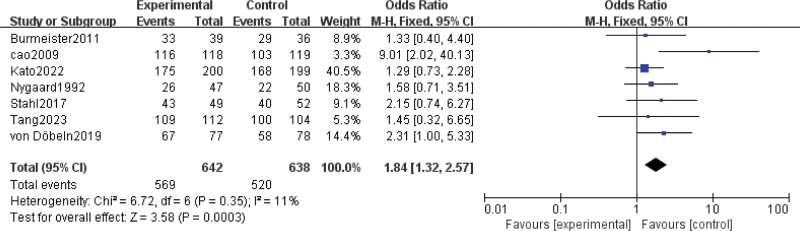
Assessment of R0 resection rate. Abbreviations: The diamond indicates best estimate of the true (pooled) outcome (with width indicating 95% CI); experimental stands for neoadjuvant chemoradiotherapy; control stands for neoadjuvant chemotherapy. Since there is low heterogeneity, a fixed-effects model is used.

Results for R0 resection rate came from 7 studies^[7,8,15,16,23–25]^ involving a total of 1280 patients. The result showed that nCRT significantly increased R0 resection rate (OR = 1.84; 95% CI: 1.32–2.57; *P* = .0003) (Fig. 4). Low heterogeneity was found among the trials (Chi^[2]^ = 6.72; df = 6 [*P* = .35]; I^2^ = 11%).

#### 3.3.5. Postoperative complications.

**Figure 5.**
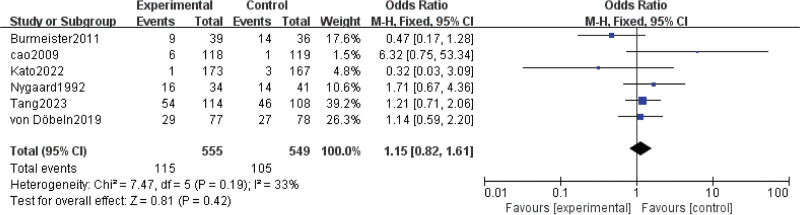
Assessment of Postoperative complications. Abbreviations: The diamond indicates best estimate of the true (pooled) outcome (with width indicating 95% CI); experimental stands for neoadjuvant chemoradiotherapy; control stands for neoadjuvant chemotherapy. Since there is moderate heterogeneity, a fixed-effects model is used.

Results for postoperative complications were extracted from 6 studies^[8,15,16,23–25]^ involving 1104 patients. The results showed that there was no difference between nCRT and nCT in the risk of postoperative complications (OR = 1.15; 95% CI: 0.82–1.61; *P* = .41) (Fig. 5). Moderate heterogeneity was found among the trials (Chi^[2]^ = 7.47; df = 5 [*P* = .19]; I^2^ = 33%).

#### 3.3.6. Postoperative mortality.

**Figure 6.**
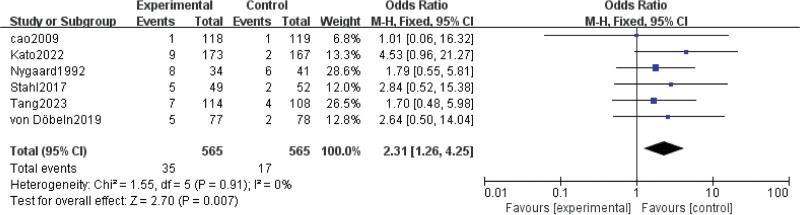
Assessment of Postoperative mortality. Abbreviations: The diamond indicates best estimate of the true (pooled) outcome (with width indicating 95% CI); experimental stands for neoadjuvant chemoradiotherapy; control stands for neoadjuvant chemotherapy. Since there is no heterogeneity, a fixed-effects model is used.

Results for postoperative mortality came from 6 studies^[7,8,15,16,24,25]^ involving a total of 1130 patients. The results showed that nCRT was associated with higher risk of postoperative mortality (OR = 2.31; 95% CI: 1.26–4.25; *P* = .007) (Fig. 6). No significant heterogeneity was found among the trials (Chi^[2]^ = 1.55; df = 5 [*P* = .91]; I^2^ = 0%).

#### 3.3.7. Grade 3 or higher adverse events.

Data for grade 3 + AEs were extracted from 4 studies^[8,15,16,23]^ involving a total of 901 patients. The results showed that nCRT resulted in severe 3 + AEs (OR = 2.21; 95% CI: 1.36–3.58; *P* = .001) (Table 2). No significant heterogeneity was found among the trials (Chi^[2]^ = 0.07; df = 1 [*P* = .79]; I^2^ = 0%). However, data for adverse events are not yet mature due to the small amount of data included.

**Table 2:** Results of grade 3 or higher adverse events.

Toxicity	Odds ratio	No. of trail	I^2^ Statistic (%)
Total	2.21 [1.36, 3.58]	2	0
Oesophagitis	1.85 [0.06, 55.37]	2	84
Pneumonitis	4.08 [0.67, 24.86]	3	0
Neutropenia	1.59 [0.70, 3.63]	3	59
Mucositis	0.73 [0.14, 3.91]	2	0
Infection	1.30 [0.42, 3.96]	2	0
Nausea/vomiting	1.56 [0.46, 5.28]	3	0
Renal impairment	0.80 [0.22, 2.91]	3	30
Cardiac disorders	1.51 [0.32, 7.06]	2	26
Anorexia	1.84 [0.98, 3.46]	2	0
Liver dysfunction	1.79 [0.37, 8.62]	2	0

### 3.4. Subgroup analysis

#### 3.4.1. Histological type.

Subgroup analysis of different histological types was performed owing to the difference of adenocarcinoma and squamous cell carcinoma in prognosis and response to chemoradiotherapy. The results of subgroup analysis suggested that both adenocarcinoma and squamous cell carcinoma could benefit from nCRT in terms of PFS and pCR. But when it came to OS and R0 resection rate, the benefits were different. nCRT significantly improved OS in esophageal squamous cell carcinoma (HR = 0.80; 95% CI: 0.68–0.98; *P* = .03), but not in esophageal adenocarcinoma (HR = 0.80; 95% CI: 0.63–1.08; *P* = .16). Moreover, nCRT was associated with higher R0 resection rate in esophageal squamous cell carcinoma (OR = 1.76; 95% CI: 1.17–2.66; *P* = .007), but not in esophageal adenocarcinoma (HR = 1.74; 95% CI: 0.79–3.86; *P* = .17) (eTable 1 in the Supplementary, http://links.lww.com/MD/L235).

#### 3.4.2. Baseline characteristics.

Subgroup analysis was performed on baseline patient characteristics, including sex, age, performance status, tumor location, cT stages and cN stages. The results showed that age, performance status, tumor location and cN stages had no effect on the efficacy of nCRT. However, women and patients with lower cT stage were more likely to benefit from nCRT (female vs male, HR = 0.47; 95% CI: 0.31–0.71; T3 vs T4, HR = 0.67; 95% CI: 0.46–0.97) (Table 3).

**Table 3:** Results of subgroup analysis for baseline characteristics in overall survival.

Outcome	Hazard ratio	No. of trail	*P* value
Female vs male	0.47 [0.31, 0.71]	2	*P* = .0004
≤60 vs > 60	0.92 [0.70, 1.21]	2	*P* = .57
Performance status 0 vs 1	0.92 [0.67, 1.26]	3	*P* = .60
Lower vs upper/middle	0.98 [0.70, 1.37]	2	*P* = .91
cT stage T3 vs T4	0.67 [0.46, 0.97]	2	*P* = .03
cN stage N0 vs N1	0.81 [0.59, 1.10]	2	*P* = .18

### 3.5. Heterogeneity, publication bias, and sensitivity analyses

The outcomes for which we identified moderate significant heterogeneity among studies were pCR (I^2^ = 46%) and postoperative complications (I^2^ = 33%). A careful review of the included studies revealed that the heterogeneity in pCR was mainly due to one trial,^[8]^ which included both adenocarcinoma and squamous cell carcinoma, possibly contributing to the heterogeneity. After removing this study, I^2^ went from 46% to 0%. Secondly, the heterogeneity of postoperative complications was mainly derived from another trial,^[23]^ and I^2^ was reduced from 33% to 3% after the removal of this study. No significant heterogeneity for all other results was found (I^2^ = 0%), except for the low heterogeneity for R0 resection rate (I^2^ = 11%). Sensitivity analysis by removing study by study found that no study affected the overall effect of the efficacy and safety endpoints, meaning that all the results were stable. Qualitative assessment was performed by assessing various measures for each individual study using the Cochrane Bias risk tool. Overall, these trials were considered to have a low-risk bias. The Egger test results showed that the potential for publication bias was low for all efficacy and safety endpoints (eTable2, http://links.lww.com/MD/L236 and eFig. 4–9 in the Supplementary, http://links.lww.com/MD/L229, http://links.lww.com/MD/L230, http://links.lww.com/MD/L231, http://links.lww.com/MD/L232, http://links.lww.com/MD/L233, http://links.lww.com/MD/L234).

## 4. Discussion

Radical operation is the most effective method for locally advanced esophageal cancer, but the effect of simple operation is not ideal. Neoadjuvant therapy can shrink the tumor before surgery, thus increasing the probability of successful radical surgery. Both nCRT and nCT are standard treatments for locally advanced esophageal cancer, which can improve the prognosis of locally advanced esophageal cancer compared with surgery alone.^[4–6,26,27]^ However, the neoadjuvant therapy for locally advanced esophageal cancer is still controversial. Current evidence does not support a survival benefit from additional radiation therapy. Previous studies and meta-analyses have shown that, compared with nCT, nCRT was associated with superior efficacy in tumor regression, but did not prolong survival time.^[12–14]^ Recently published clinical trials have shown that nCRT is still a promising treatment option.^[15,16]^ Therefore, we conducted an updated meta-analysis to reevaluate the efficacy and safety of nCRT and nCT in locally advanced esophageal cancer. The results showed that nCRT could significantly improve PFS, OS, pCR and R0 resection rate. Unlike previous meta-analyses, our meta-analysis supported the survival benefit of additional radiotherapy, and performed subgroup analyses to investigate the effect of histological type and baseline characteristics on the efficacy of nCRT. Guo et al ‘s meta-analysis found that nCRT could prolong survival, but the reliability of its conclusions was low due to the large number of retrospective studies included in this meta-analysis.^[11]^ Another network meta-analysis by Kelvin et al found that in the direct comparison between nCRT and nCT, there was no significant statistical difference in OS, but indirect comparison showed the opposite result.^[10]^ Our meta-analysis focused only on randomized controlled trials that compared head-to-head nCRT to nCT, and updated recent studies. Moreover, group comparisons were reported using HR for OS and PFS, which allowed for a more comprehensive assessment and reduced bias.

Pathological complete response is a recognized predictor of survival after neoadjuvant therapy,^[28–30]^ and complete histopathological responses at the primary site also indicate that chemotherapy responds to peripheral micrometastases, which can partly explain why pCR is a prognostic marker of survival. Studies back this up. The NEOCRTEC5010 trial showed that patients achieving pCR was associated with longer OS than those who did not (HR, 2.70; 95% CI: 1.48–4.92; *P* = .001).^[26]^ In the CMISG1701 study, the pCR of nCRT was significantly higher than that of nCT (27.7% vs 2.9%).^[15]^ In addition, the gap was even greater in the JCOG1109 trial, with 77 patients achieving pCR in nCRT compared to only 4 patients achieving pCR in nCT,^[16]^ indicating that additional radiotherapy was associated with superior efficacy in tumor regression. Our meta-analysis also found that nCRT significantly improved pCR (OR = 13.00; 95% CI: 7.82–21.61). Interestingly, only one of the included studies showed a statistical difference in OS.^[24]^ No significant OS benefit was found in the remaining 6 studies.^[7,8,15,16,23,25]^ One possible reason for this result was that additional radiation therapy increased tumor regression, but the lymphatic dissection and high R0 resection rate may have eliminated this benefit. Even so, most studies also show a tendency to prolong the OS. The pooled results of our meta-analysis found superior efficacy associated with nCRT in OS.

Due to biological differences between adenocarcinoma and squamous cell carcinoma, sensitivity to neoadjuvant therapy is also different.^[5,6,31–33]^ We performed a subgroup analysis to explore this difference. The results showed that nCRT significantly prolonged OS in squamous cell carcinoma, but not in adenocarcinoma, indicating that squamous cell carcinoma is more sensitive to chemoradiotherapy than adenocarcinoma. In addition, the recurrence of adenocarcinoma is usually dominated by peripheral metastasis, owing to the high risk of latent systemic metastasis of adenocarcinoma.^[34,35]^ This insidious systemic disease is difficult to improve overall survival by local radiotherapy. In addition, we performed a subgroup analysis to explore the effect of baseline characteristics on nCRT. The result showed that women had a better prognosis than men. Similarly, A retrospective analysis by Kauppila et al involving 1816 patients with esophageal cancer found that women with esophageal squamous cell carcinoma was associated with lower overall all-cause mortality compared with men (HR = 0.73, 95%CI 0.63–0.85), but the benefit was limited to women over 55 years old. However, no similar difference was observed in adenocarcinoma.^[36]^ Likewise, another study also found that sex was a prognostic factor for esophageal squamous cell carcinoma, but not adenocarcinoma.^[37]^ It not clear why women have a better prognosis. One possible biological mechanism for the sex difference is the effect of estrogen, which inhibits the growth of cancer cells.^[38–40]^ In addition, hormone replacement therapy may reduce the risk of esophageal squamous cell cancer. Further research is expected to explain this phenomenon.

For adverse events, our meta-analysis found that nCRT was associated with more severe grade 3 + AEs. But because of the small number of trials included, the data are still premature. Postoperative complications and death are one of the reasons that limit the use of radiotherapy and may offsetting the survival benefits of additional radiotherapy. Our meta-analysis found that additional radiotherapy did not lead to higher postoperative complications, but increased the risk of postoperative mortality. The higher postoperative mortality in patients undergoing neoadjuvant surgery may be partly due to the trauma caused by open esophagectomy.^[41,42]^ Minimally invasive esophagectomy (MIE) has become the mainstream minimally invasive surgery for resection of esophageal cancer worldwide due to its advantages of low trauma, rapid recovery and equal tumor survival rate.^[43]^ In the CMISG1701 trial, minimally invasive esophagectomy was performed after nCRT and nCT. Unlike previous studies, no significant differences were found between the 2 groups in total complications, severe complications, mortality at 90 days after surgery, or treatment-related deaths at 1 year after surgery.^[15,44]^ A retrospective study found that MIE reduced complications and postoperative mortality without affecting overall survival compared with open esophagectomy.^[43]^

Neoadjuvant therapy is blooming and has become the most active field in the study of locally advanced esophageal cancer. However, 30% to 40% of patients still have postoperative recurrence after neoadjuvant therapy, especially patients who have not received pCR, with a higher recurrence rate.^[45]^ At present, immunotherapy is a hot research topic. KEYNOTE-590 suggests the potential of immunotherapy combined with chemotherapy in neoadjuvant therapy for locally advanced esophageal cancer.^[46]^ A recent meta-analysis included 27 clinical trials evaluating neoadjuvant immunotherapy with a pooled pCR of 31.4%.^[47]^ According to the results of meta-analysis, the PCR of nCT combined with immunotherapy did not reach that of nCRT. In 2019, a single-arm, single-center phase II clinical study was conducted in South Korea. Patients with esophageal squamous cell carcinoma were enrolled and received nCRT (total radiation dose 44.1Gy/12fx) combined with pembrolizumab and surgery 6 to 8 weeks after nCRT. Pembrolizumab was maintained for 2 years after surgery or disease progression. The results showed that the pCR was 46.1% and the postoperative mortality was 7.7% (2/26).^[48]^ ASCO recently published a prospective single-center single-arm Phase Ib clinical study to explore the safety and efficacy of short-course nCRT (total radiation dose 30Gy/12fx) combined with toripalimab in patients with locally advanced resectable esophageal squamous cell carcinoma. The results showed that 20 of the 23 patients underwent surgery, and 11 patients achieved pCR (55%).^[49]^ These trials indicate that nCRT combined with immunotherapy is a promising neoadjuvant therapy, but most of them are phase I-II clinical trials with small sample sizes, so more large randomized controlled trials are needed for further verification in the future.

This meta-analysis has several strengths including the prospective registration of the systematic review protocol and low heterogeneity in most efficacy end point. Additionally, group comparisons were reported using HR for OS and PFS. Moreover, our meta-analysis represents the full range of randomized evidence for the effect of adding radiation therapy to preoperative chemotherapy in patients with locally advanced esophageal cancer. However, there are some limitations to our meta-analysis. First, the included clinical trials spanned a large number of years. Second, subgroup analysis of baseline characteristics and adverse event data are not yet available due to insufficient data. Third, part of the data is calculated according to the previous method.

## 5. Conclusions

Our meta-analysis found that superior efficacy associated with nCRT compared with nCT in both tumor regression and prolonged survival, but increased the risk of postoperative mortality and grade 3 + AEs. Survival benefits were different for adenocarcinoma and squamous cell carcinoma. Squamous cell carcinoma was more likely to benefit from nCRT than adenocarcinoma.

## Author contributions

**Conceptualization:** Zhanpeng Liang, Ting Chen, Cantu Fang.

**Data curation:** Zhanpeng Liang, Ting Chen.

**Formal analysis:** Zhanpeng Liang, Ting Chen.

**Funding acquisition:** Huiqin Lai, Cantu Fang.

**Investigation:** Wenxia Li, Huiqin Lai, Luzhen Li, Huatang Zhang, Cantu Fang.

**Methodology:** Zhanpeng Liang, Ting Chen, Wenxia Li, Luzhen Li, Huatang Zhang.

**Project administration:** Zhanpeng Liang, Ting Chen, Wenxia Li, Jiaming Wu.

**Resources:** Wenxia Li, Huiqin Lai, Jiaming Wu, Cantu Fang.

**Software:** Zhanpeng Liang, Ting Chen, Wenxia Li.

**Supervision:** Huiqin Lai, Luzhen Li, Huatang Zhang, Cantu Fang.

**Validation:** Huiqin Lai, Luzhen Li, Jiaming Wu, Huatang Zhang, Cantu Fang.

**Visualization:** Jiaming Wu, Huatang Zhang, Cantu Fang.

**Writing – original draft:** Zhanpeng Liang, Ting Chen.

**Writing – review & editing:** Luzhen Li, Jiaming Wu, Huatang Zhang, Cantu Fang.

## Supplementary Material
























