# Supplementary material for: Efficacy and safety of neoadjuvant chemoradiotherapy versus neoadjuvant chemotherapy in locally advanced esophageal cancer: An updated meta-analysis
Source: Medicine (Baltimore). 2024 Jan 19;103(3):e36785. doi: 10.1097/MD.0000000000036785 (PMC10798774; doi:10.1097/MD.0000000000036785)
Supplement: Supplementary file 1 [file medi-103-e36785-s001.docx]

**eMethods.** Search Strategy

**PubMed < updated to 2023-04-22> Search Strategy (733)**

**#1 "Chemoradiotherapy, Adjuvant"[Mesh]**

**#2 Neoadjuvant Chemoradiotherapy[Title/Abstract] OR Chemoradiotherapy, Neoadjuvant[Title/Abstract] OR Neoadjuvant Chemoradiotherapies[Title/Abstract] OR Neoadjuvant Chemoradiation Therapy[Title/Abstract] OR Chemoradiation Therapy, Neoadjuvant[Title/Abstract] OR Neoadjuvant Chemoradiation Therapies[Title/Abstract] OR Therapy, Neoadjuvant Chemoradiation[Title/Abstract] OR Neoadjuvant Chemoradiation Treatment[Title/Abstract] OR Chemoradiation Treatment, Neoadjuvant[Title/Abstract] OR Neoadjuvant Chemoradiation Treatments[Title/Abstract] OR Treatment, Neoadjuvant Chemoradiation[Title/Abstract] OR Neoadjuvant Chemoradiation[Title/Abstract] OR Chemoradiation, Neoadjuvant[Title/Abstract] OR Neoadjuvant Chemoradiations[Title/Abstract] OR Neoadjuvant Radiotherapy[Title/Abstract] OR Neoadjuvant Radiotherapies[Title/Abstract] OR Radiotherapy, Neoadjuvant[Title/Abstract] OR Neoadjuvant Radiation[Title/Abstract] OR Radiation, Neoadjuvant[Title/Abstract] OR Pre-Operative Radiotherapy[Title/Abstract] OR chemoradiation therapy[Title/Abstract] OR chemoradiotherapy[Title/Abstract] OR radiochemotherapy[Title/Abstract]**

**#3 #1 OR #2**

**#4 "Chemotherapy, Adjuvant"[Mesh]**

**#5 Neoadjuvant Chemotherapy[Title/Abstract] OR Chemotherapy, Neoadjuvant[Title/Abstract] OR Neoadjuvant Chemotherapies[Title/Abstract] OR Neoadjuvant Chemotherapy Treatment[Title/Abstract] OR Chemotherapy Treatment, Neoadjuvant[Title/Abstract] OR Neoadjuvant Chemotherapy Treatments[Title/Abstract] OR Treatment, Neoadjuvant Chemotherapy[Title/Abstract] OR Preoperative chemotherapy[Title/Abstract] OR pre-operative chemotherapy[Title/Abstract] OR neoadjuvant treatment[Title/Abstract]**

**#6 #4 OR #5**

**#7 "Surgical Procedures, Operative"[Mesh] OR "General Surgery"[Mesh] OR "surgery" [Subheading] OR "Esophagectomy"[Mesh]**

**#8 Operative Procedures[Title/Abstract] OR Operative Procedure[Title/Abstract] OR Procedure, Operative[Title/Abstract] OR Procedures, Operative[Title/Abstract] OR Surgical Procedure, Operative[Title/Abstract] OR Operative Surgical Procedures[Title/Abstract] OR Procedure, Operative Surgical[Title/Abstract] OR Procedures, Operative Surgical[Title/Abstract] OR Surgical Procedures[Title/Abstract] OR Procedure, Surgical[Title/Abstract] OR Procedures, Surgical[Title/Abstract] OR Surgical Procedure[Title/Abstract] OR Operative Surgical Procedure[Title/Abstract] OR Surgery, Ghost[Title/Abstract] OR Ghost Surgery[Title/Abstract] OR Esophagectomy[Title/Abstract]**

**#9 #7 OR #8**

**#10 "Esophageal Neoplasms"[Mesh]**

**#11**  **Esophageal Neoplasm[Title/Abstract] OR Neoplasm, Esophageal[Title/Abstract] OR Esophagus Neoplasm[Title/Abstract] OR Esophagus Neoplasms[Title/Abstract] OR Neoplasm, Esophagus[Title/Abstract] OR Neoplasms, Esophagus[Title/Abstract] OR Neoplasms, Esophageal[Title/Abstract] OR Cancer of Esophagus[Title/Abstract] OR Cancer of the Esophagus[Title/Abstract] OR Esophagus Cancer[Title/Abstract] OR Cancer, Esophagus[Title/Abstract] OR Cancers, Esophagus[Title/Abstract] OR Esophagus Cancers[Title/Abstract] OR Esophageal Cancer[Title/Abstract] OR Cancer, Esophageal[Title/Abstract] OR Cancers, Esophageal[Title/Abstract] OR Esophageal Cancers[Title/Abstract]**

**#12 #10 OR #11**

**#13 #3 OR #6 OR #9 OR #12**

**Embase < updated to 2023-04-22> Search Strategy（1357）**

**#1 'esophagus cancer'/exp**

**#2 'esophageal neoplasm':ab,ti OR 'neoplasm, esophageal':ab,ti OR 'esophagus neoplasm':ab,ti OR 'esophagus neoplasms':ab,ti OR 'neoplasm, esophagus':ab,ti OR 'neoplasms, esophagus':ab,ti OR 'neoplasms, esophageal':ab,ti OR 'cancer of esophagus':ab,ti OR 'cancer of the esophagus':ab,ti OR 'esophagus cancer':ab,ti OR 'cancer, esophagus':ab,ti OR 'cancers, esophagus':ab,ti OR 'esophagus cancers':ab,ti OR 'esophageal cancer':ab,ti OR 'cancer, esophageal':ab,ti OR 'cancers, esophageal':ab,ti OR 'esophageal cancers':ab,ti**

**#3 #1 OR #2**

**#4 'surgery'/exp**

**#5 'operative procedures':ab,ti OR 'operative procedure':ab,ti OR 'procedure, operative':ab,ti OR 'procedures, operative':ab,ti OR 'surgical procedure, operative':ab,ti OR 'operative surgical procedures':ab,ti OR 'procedure, operative surgical':ab,ti OR 'procedures, operative surgical':ab,ti OR 'surgical procedures':ab,ti OR 'procedure, surgical':ab,ti OR 'procedures, surgical':ab,ti OR 'surgical procedure':ab,ti OR 'operative surgical procedure':ab,ti OR 'surgery, ghost':ab,ti OR 'ghost surgery':ab,ti OR esophagectomy:ab,ti**

**#6 #4 OR #5**

**#7 'neoadjuvant chemotherapy'/exp**

**#8 'neoadjuvant chemotherapy':ab,ti OR 'chemotherapy, neoadjuvant':ab,ti OR 'neoadjuvant chemotherapies':ab,ti OR 'neoadjuvant chemotherapy treatment':ab,ti OR 'chemotherapy treatment, neoadjuvant':ab,ti OR 'neoadjuvant chemotherapy treatments':ab,ti OR 'treatment, neoadjuvant chemotherapy':ab,ti OR 'preoperative chemotherapy':ab,ti OR 'pre-operative chemotherapy':ab,ti OR 'neoadjuvant treatment':ab,ti**

**#9 #7 OR #8**

**#10 'neoadjuvant chemoradiotherapy'/exp**

**#11 'neoadjuvant chemoradiotherapy':ab,ti OR 'chemoradiotherapy, neoadjuvant':ab,ti OR 'neoadjuvant chemoradiotherapies':ab,ti OR 'neoadjuvant chemoradiation therapy':ab,ti OR 'chemoradiation therapy, neoadjuvant':ab,ti OR 'neoadjuvant chemoradiation therapies':ab,ti OR 'therapy, neoadjuvant chemoradiation':ab,ti OR 'neoadjuvant chemoradiation treatment':ab,ti OR 'chemoradiation treatment, neoadjuvant':ab,ti OR 'neoadjuvant chemoradiation treatments':ab,ti OR 'treatment, neoadjuvant chemoradiation':ab,ti OR 'neoadjuvant chemoradiation':ab,ti OR 'chemoradiation, neoadjuvant':ab,ti OR 'neoadjuvant chemoradiations':ab,ti OR 'neoadjuvant radiotherapy':ab,ti OR 'neoadjuvant radiotherapies':ab,ti OR 'radiotherapy, neoadjuvant':ab,ti OR 'neoadjuvant radiation':ab,ti OR 'radiation, neoadjuvant':ab,ti OR 'pre-operative radiotherapy':ab,ti OR 'chemoradiation therapy':ab,ti OR chemoradiotherapy:ab,ti OR radiochemotherapy:ab,ti**

**#12 #10 OR #11**

**#13 #3 AND #6 AND #9 AND #12**

**Cochrane library < updated to 2023-04-22>（223）**

**#1 MeSH descriptor: [Esophageal Neoplasms] explode all trees**

**#2 (Esophageal Neoplasm OR Neoplasm, Esophageal OR Esophagus Neoplasm OR Esophagus Neoplasms OR Neoplasm, Esophagus OR Neoplasms, Esophagus OR Neoplasms, Esophageal OR Cancer of Esophagus OR Cancer of the Esophagus OR Esophagus Cancer OR Cancer, Esophagus OR Cancers, Esophagus OR Esophagus Cancers OR Esophageal Cancer OR Cancer, Esophageal OR Cancers, Esophageal OR Esophageal Cancers):ti,ab,kw**

**#3 #1 OR #2**

**#4 MeSH descriptor: [Specialties, Surgical] explode all trees**

**#5 (Operative Procedures OR Operative Procedure OR Procedure, Operative OR Procedures, Operative OR Surgical Procedure, Operative OR Operative Surgical Procedures OR Procedure, Operative Surgical OR Procedures, Operative Surgical OR Surgical Procedures OR Procedure, Surgical OR Procedures, Surgical OR Surgical Procedure OR Operative Surgical Procedure OR Surgery, Ghost OR Ghost Surgery OR Esophagectomy):ti,ab,kw**

**#6 #4 OR #5**

**#7 MeSH descriptor: [Neoadjuvant Therapy] explode all trees**

**#8 (Neoadjuvant Chemotherapy OR Chemotherapy, Neoadjuvant OR Neoadjuvant Chemotherapies OR Neoadjuvant Chemotherapy Treatment OR Chemotherapy Treatment, Neoadjuvant OR Neoadjuvant Chemotherapy Treatments OR Treatment, Neoadjuvant Chemotherapy OR Preoperative chemotherapy OR pre-operative chemotherapy OR neoadjuvant treatment):ti,ab,kw**

**#9 #7 OR #8**

**#10 MeSH descriptor: [Neoadjuvant Therapy] explode all trees**

**#11 (Neoadjuvant Chemoradiotherapy OR Chemoradiotherapy, Neoadjuvant OR Neoadjuvant Chemoradiotherapies OR Neoadjuvant Chemoradiation Therapy OR Chemoradiation Therapy, Neoadjuvant OR Neoadjuvant Chemoradiation Therapies OR Therapy, Neoadjuvant Chemoradiation OR Neoadjuvant Chemoradiation Treatment OR Chemoradiation Treatment, Neoadjuvant OR Neoadjuvant Chemoradiation Treatments OR Treatment, Neoadjuvant Chemoradiation OR Neoadjuvant Chemoradiation OR Chemoradiation, Neoadjuvant OR Neoadjuvant Chemoradiations OR Neoadjuvant Radiotherapy OR Neoadjuvant Radiotherapies OR Radiotherapy, Neoadjuvant OR Neoadjuvant Radiation OR Radiation, Neoadjuvant OR Pre-Operative Radiotherapy OR chemoradiation therapy OR chemoradiotherapy OR radiochemotherapy):ti,ab,kw**

**#12 #10 OR #11**

**#13 #3 AND #6 AND #9 AND #12**
