# Supplementary figures and images for: Efficacy and safety of neoadjuvant chemoradiotherapy versus neoadjuvant chemotherapy in locally advanced esophageal cancer: An updated meta-analysis
Source: Medicine (Baltimore). 2024 Jan 19;103(3):e36785. doi: 10.1097/MD.0000000000036785 (PMC10798774; doi:10.1097/MD.0000000000036785)

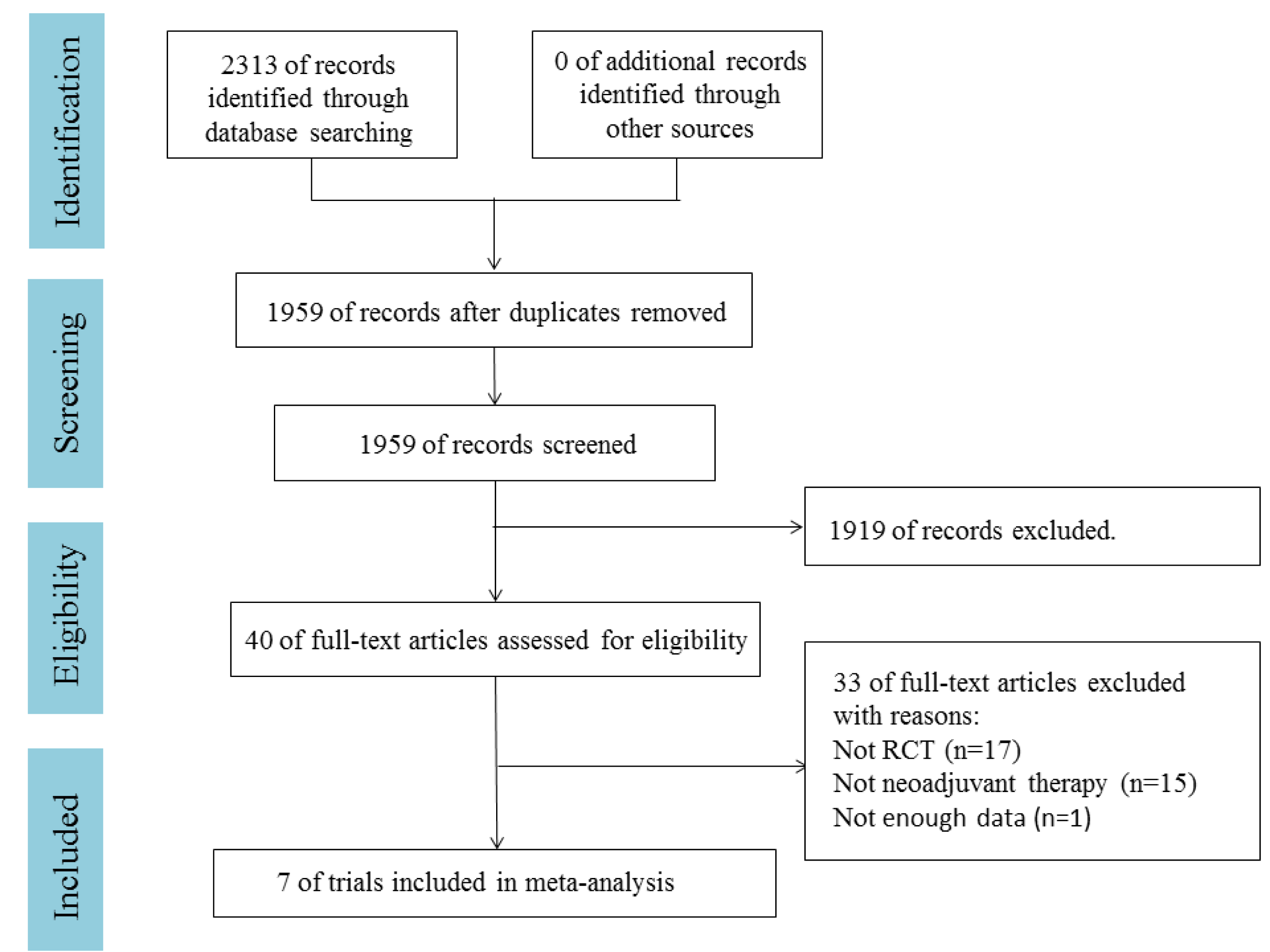

Supplement: Supplementary file 2 [file medi-103-e36785-s002.tif]

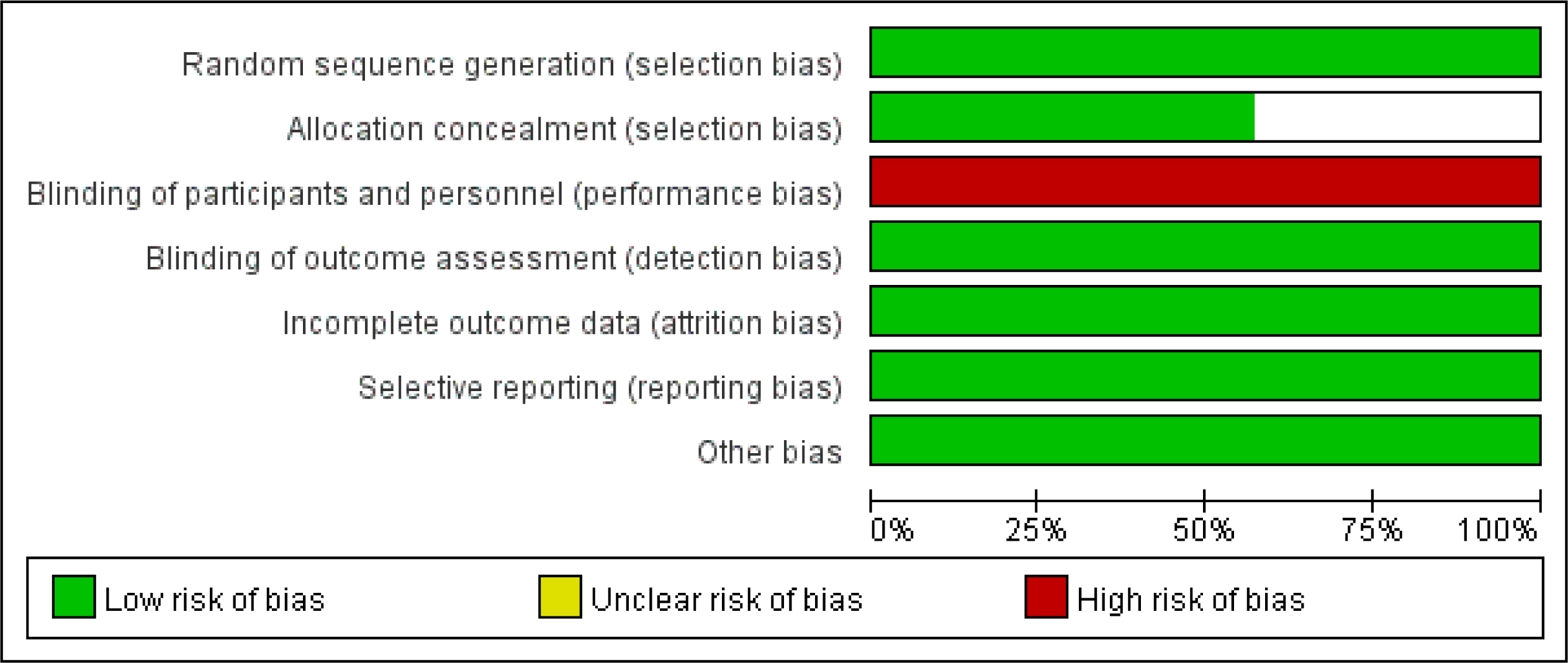

Supplement: Supplementary file 3 [file medi-103-e36785-s003.tif]

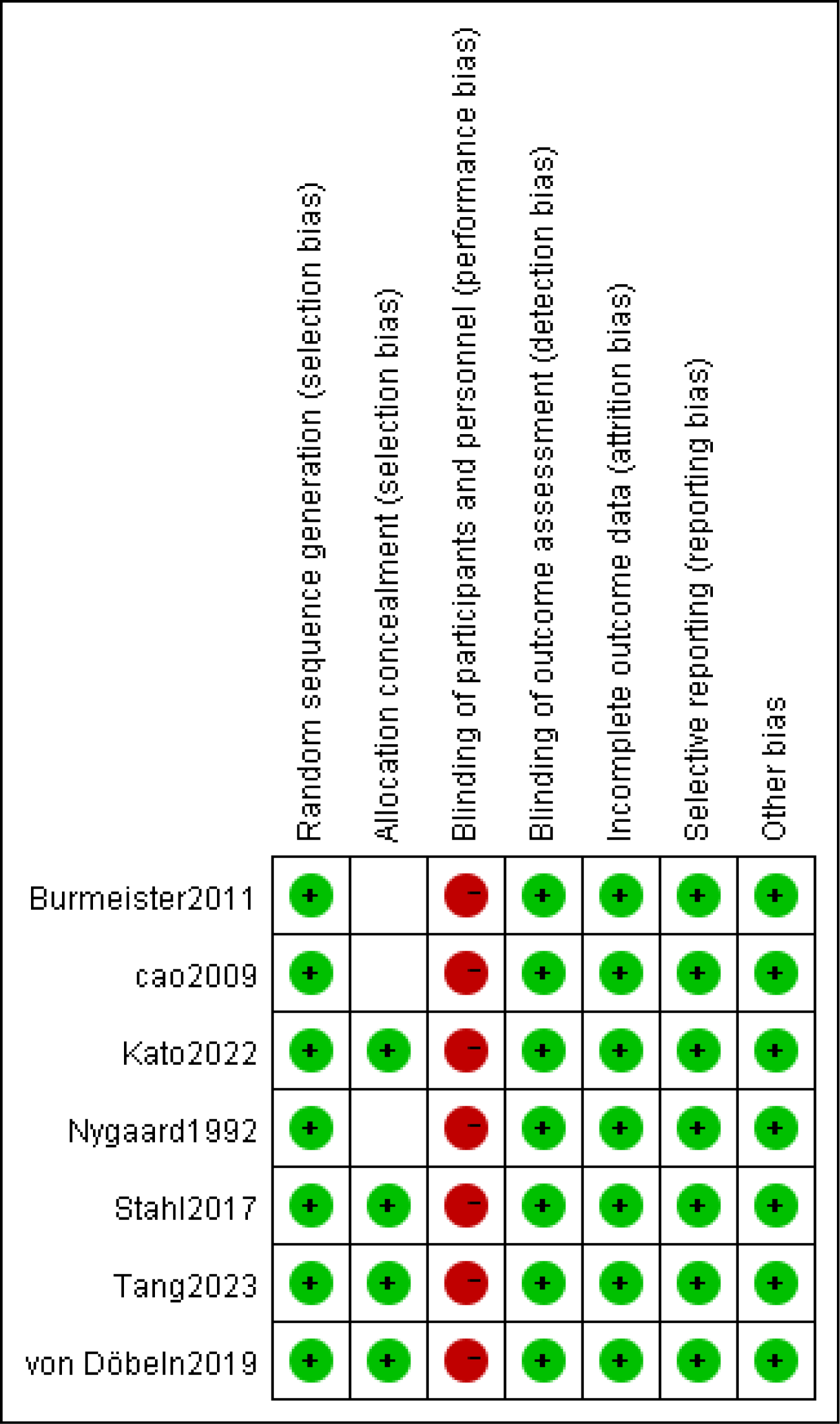

Supplement: Supplementary file 4 [file medi-103-e36785-s004.tif]

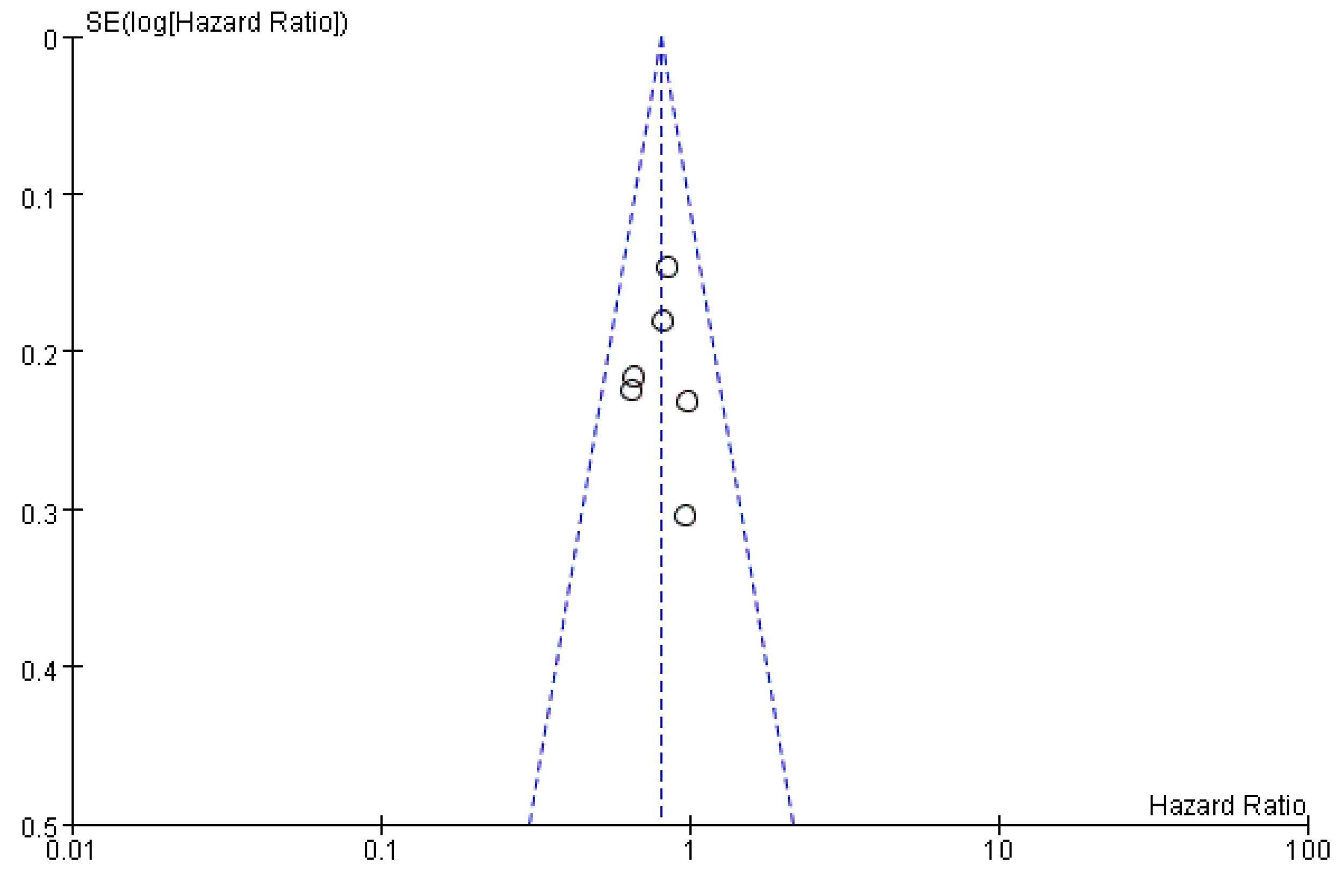

Supplement: Supplementary file 7 [file medi-103-e36785-s007.tif]

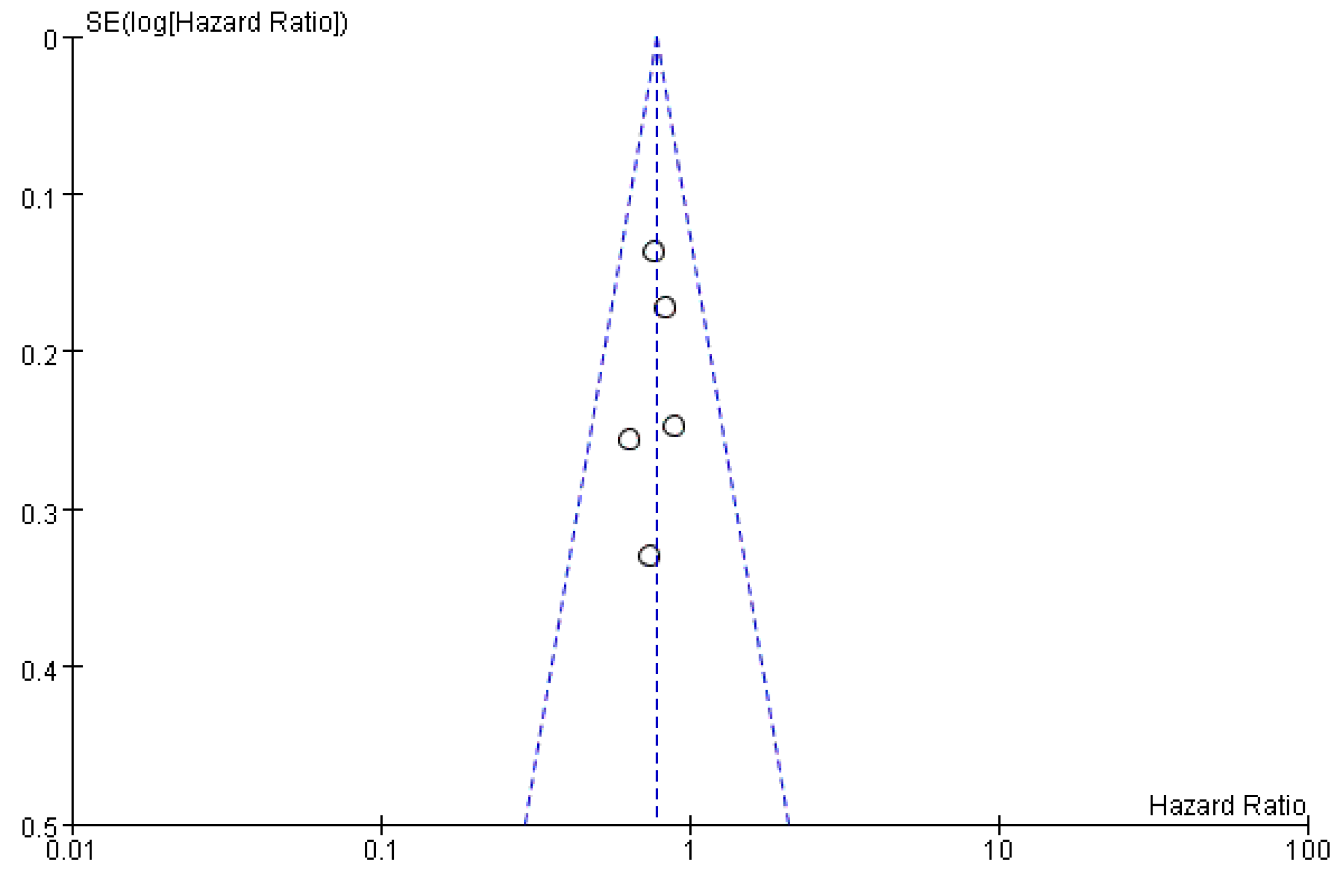

Supplement: Supplementary file 8 [file medi-103-e36785-s008.tif]

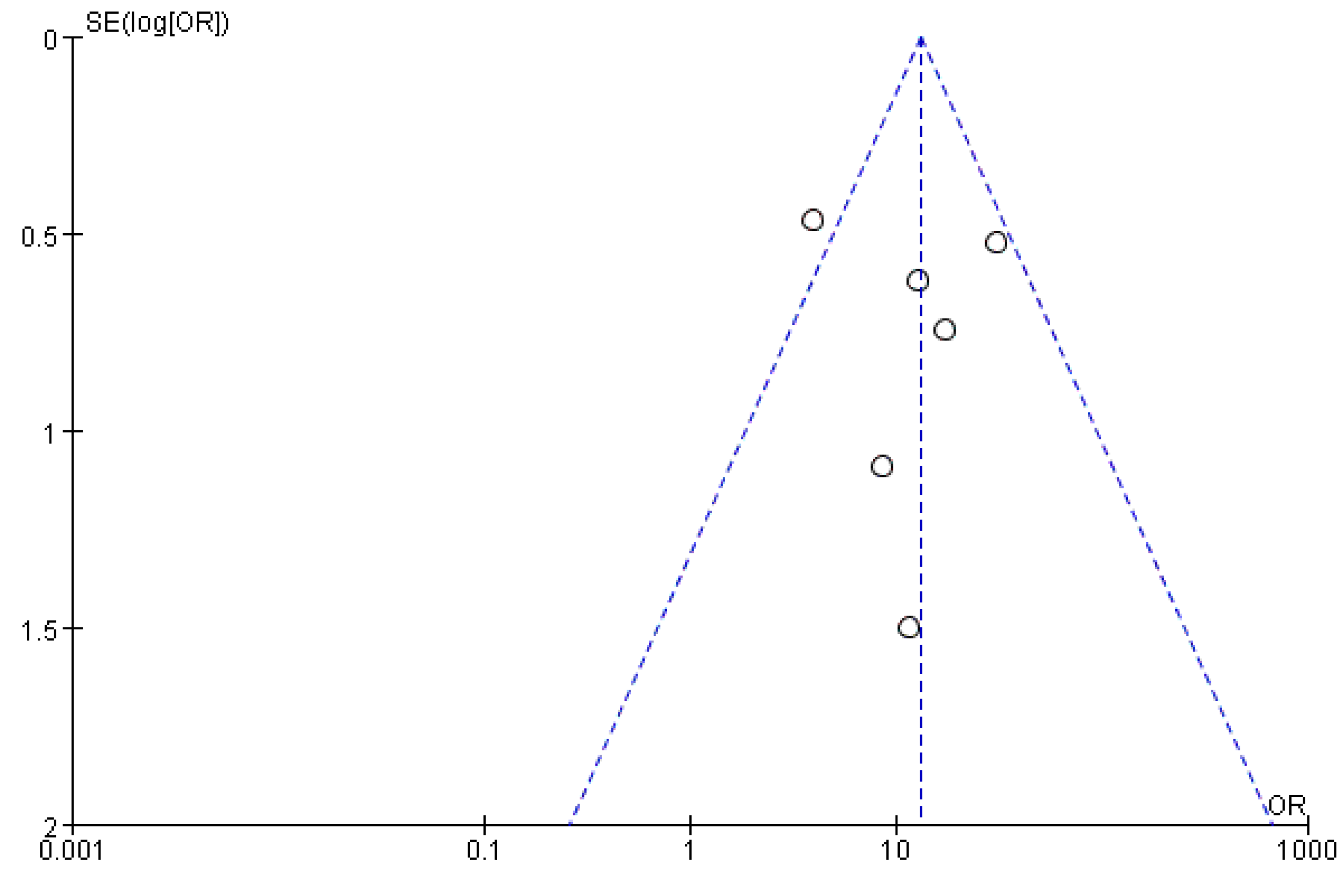

Supplement: Supplementary file 9 [file medi-103-e36785-s009.tif]

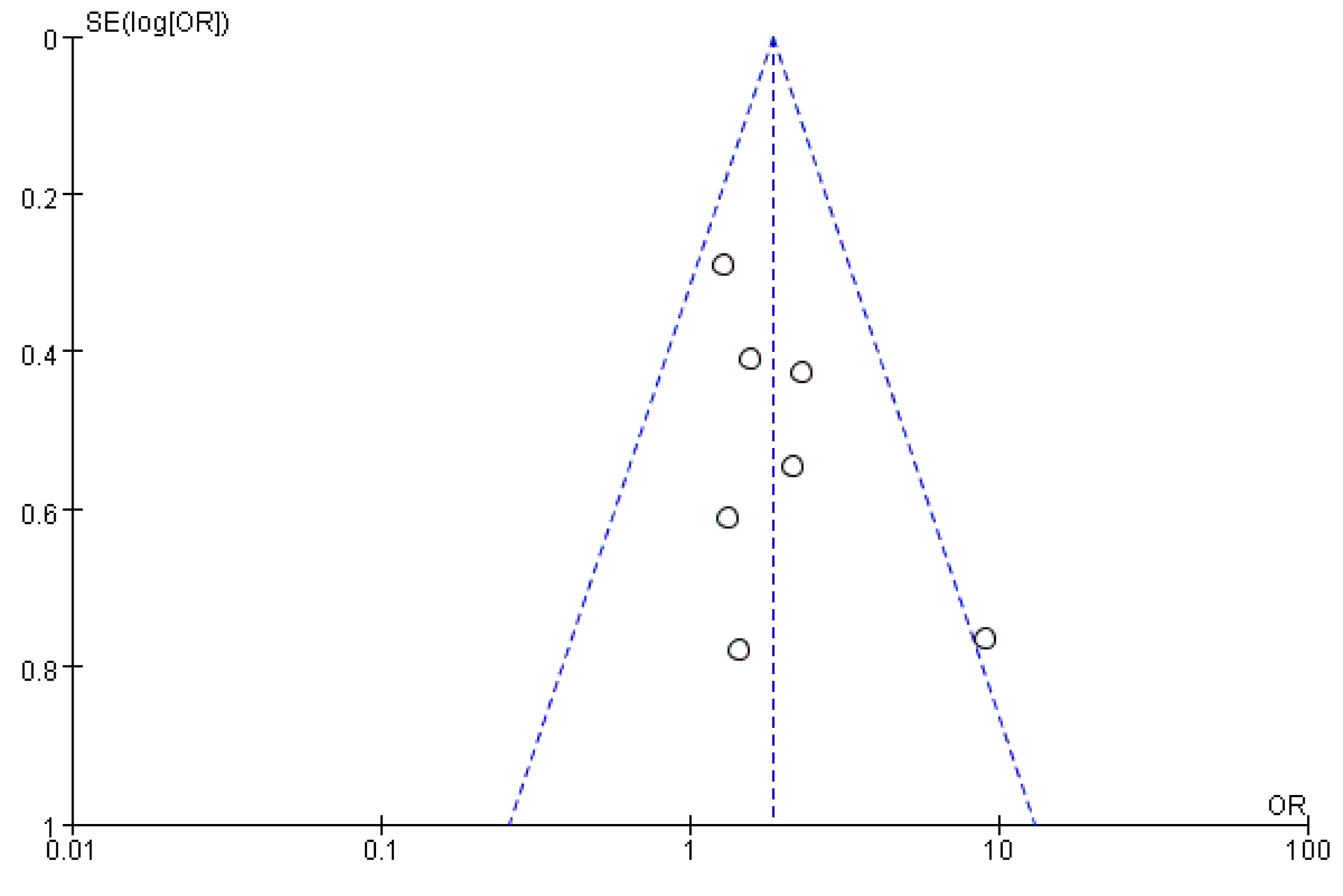

Supplement: Supplementary file 10 [file medi-103-e36785-s010.tif]

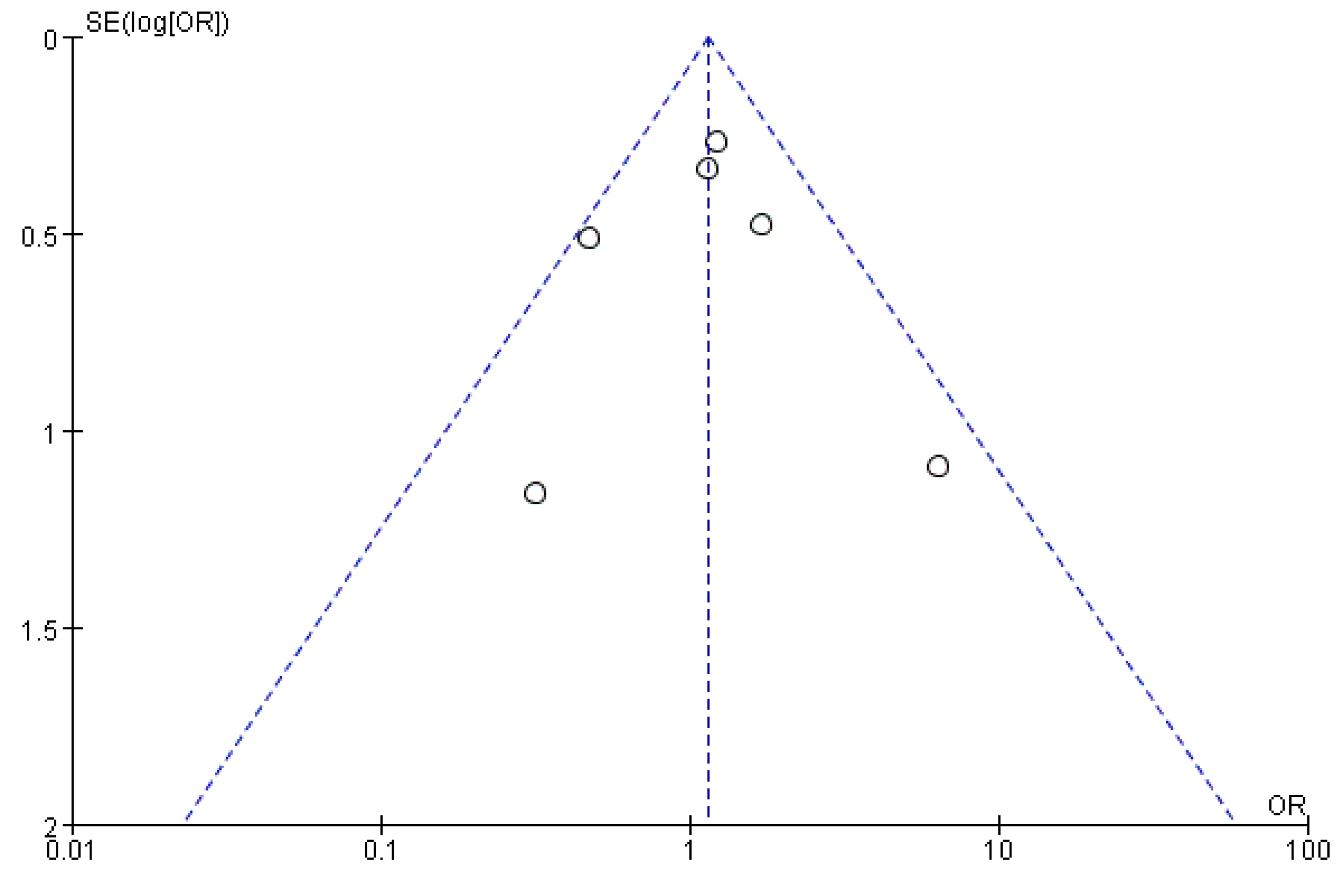

Supplement: Supplementary file 11 [file medi-103-e36785-s011.tif]

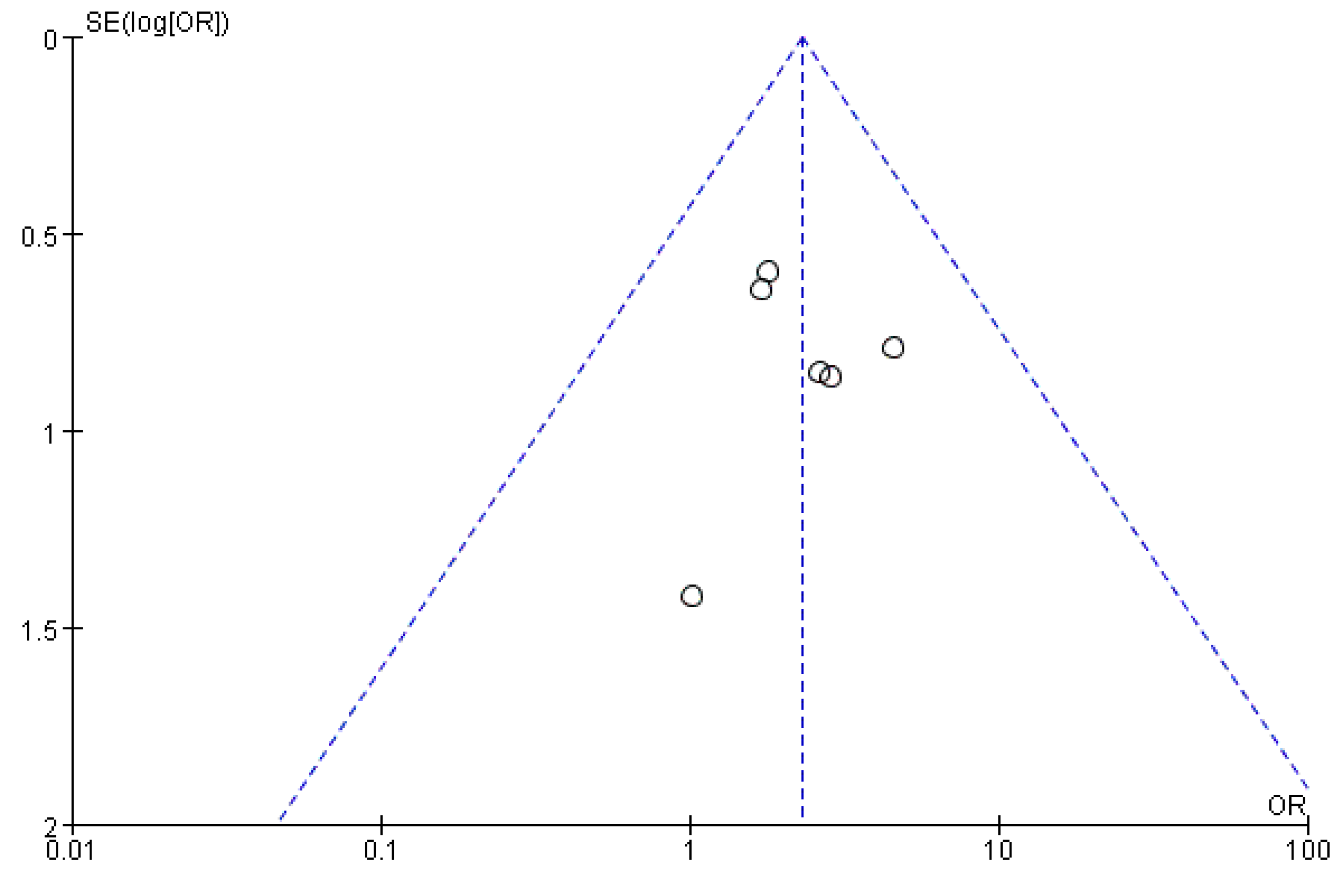

Supplement: Supplementary file 12 [file medi-103-e36785-s012.tif]
