# Supplementary material for: Efficacy and safety of neoadjuvant chemoradiotherapy versus neoadjuvant chemotherapy in locally advanced esophageal cancer: An updated meta-analysis
Source: Medicine (Baltimore). 2024 Jan 19;103(3):e36785. doi: 10.1097/MD.0000000000036785 (PMC10798774; doi:10.1097/MD.0000000000036785)
Supplement: Supplementary file 5 [file medi-103-e36785-s005.docx]

**eTable1.** Results of subgroup analysis for histological type

| **Outcome** | **Squamous cell carcinoma** | **No. of trail** | **adenocarcinoma** | **No. of trail** |
| --- | --- | --- | --- | --- |
| OS (HR and 95%CI) | 0.81 [0.67, 0.98] | 4 | 0.82 [0.63, 1.08] | 3 |
| PFS (HR and 95%CI) | 0.79 [0.64, 0.98] | 2 | 0.68 [0.46, 1.00] | 2 |
| pCR (OR and 95%CI) | 16.18 [8.08, 32.41] | 3 | 9.60 [1.71, 53.86] | 2 |
| R0 resection rate  (OR and 95%CI) | 1.76 [1.17, 2.66] | 4 | 1.74 [0.79, 3.86] | 2 |

Abbreviations: OS: overall survival, PFS: progression-free survival; pCR: pathological complete response; HR: hazard ratio; OR: odds ratio
