# Supplementary material for: Efficacy and safety of neoadjuvant chemoradiotherapy versus neoadjuvant chemotherapy in locally advanced esophageal cancer: An updated meta-analysis
Source: Medicine (Baltimore). 2024 Jan 19;103(3):e36785. doi: 10.1097/MD.0000000000036785 (PMC10798774; doi:10.1097/MD.0000000000036785)
Supplement: Supplementary file 6 [file medi-103-e36785-s006.docx]

**eTable2.** Results of Egger's test

| **Outcome** | **P value** | **Publication bias** |
| --- | --- | --- |
| Overall survival | 0.944 | No |
| Progression-free survival | 0.740 | No |
| Pathological complete response | 0.591 | No |
| R0 resection rate | 0.174 | No |
| Postoperative complications | 0.960 | No |
| Postoperative mortality | 0.992 | No |
